# On the System of the Great Sympathetic Nerve

**Published:** 1847-10-01

**Authors:** 


### On the System of the Great Sympathetic Nerve. First Part. By *C. Radclyffe Hall*, M.D. Republished from the Edinburgh Medical and Surgical Journal. 1847.

An additional attempt to solve the enigma of the Great Sympathetic is not a very promising topic for the generality of medical readers. We have, however, no wish either to underrate the importance of the subject or to depreciate the merits of Dr. C. R. Hall's memoir; but if this paper had not been brought more directly under our notice in the shape of a separate pamphlet, it might, without any injury to science, have been left in the pages of the periodical in which it originally appeared, for the consideration of the professed physiologist, to whose acumen the experiments and reasonings of the author are more particularly suited. A few extracts will enable our readers to understand the general views here promulgated; those who require more minute information, must refer to the work itself.

We have always ourselves selected the cephalic ganglia as the type of the great sympathetic; because here, as in so many analogous instances, Nature has offered for our investigation an analysis of what, in other parts of the same system, is obscured and involved. For instance, in these scattered ganglionic masses the observer, owing to the natural severation of the motor and sentient elements of the cranial nerves, which in the case of the spinal nerves are bound up together, is enabled to detect a fundamental fact connected with the sympathetic, namely, that its ganglia receive from the cerebro-spinal system both motor and sentient branches; he can further perceive that each of these isolated nodules of gray matter is brought into anatomical relation with the rest of the sympathetic by special filaments of communication, often running a long and tortuous course to effect the junction. Similar reasons, joined to the comparative facility of access and to the ease with which the results of experiment produced on the eye can be observed, have induced Dr. C. R. Hall to choose the ophthalmic ganglion for investigation—" the lenticular ganglion represents the ganglionic system in miniature; what can be proved with respect to it, may justly be taken as a safe index of the rest."

The following observations on the varying states of the pupil are worthy of attention:—

" The pupil after death in every animal is of the medium size natural during life. The exceptions are numerous, and depend upon the state of the iris at the time of death, and upon the action of external agents after death. If the pupil is very large when death occurs, as in slow death from hanging, or prussic acid, it will retain this size for some time after death; as the body cools, the pupil lessens, but is seldom reduced to the medium size. But when death from prussic acid is instantaneous, the largely dilated pupils will contract in a few minutes after death to less than the medium size, and afterwards enlarge again. The varying degree of contraction of the *sphincter iridis* at the time of coagulation of the blood, (which *does* take place, at least in small vessels, after poisoning by prussic acid,) may perhaps account for this. If death occurs with the pupils contracted, these enlarge after a time, but do not generally attain the ordinary medium size. In practising operations on the eye of the human subject after death before the animal heat has vanished, a dilated pupil always contracts as the aqueous humour escapes. This, like the motion of a narcotized iris in extracting the lens for cataract, and like the contraction of the previously dilated pupil of a drowned kitten on exposing the animal to the heat of a furnace, in an experiment performed by Haller, is probably due to physical influence." P. 3.

The author has corrected some errors of former anatomists, such as that the ophthalmic ganglion is absent in the rodentia; on the contrary, he found it beneath the optic nerve, but so minute as to require great care in the dissection, in the rabbit, squirrel and guinea-pig. The conclusions drawn from the various experiments upon the ophthalmic ganglion and the nerves connected with it are thus set forth:—

" 1. The third nerve is the only direct motor nerve for contraction of the pupil in dogs and cats.

" 2. The action of the third nerve, as far as concerns the iris, is mainly under the controul of the visual nervous tract.

" 3. As both the third nerve and some portion of the visual tract must inevitably be injured in performing the experiment of dividing the fifth nerve after Magendie's plan, the experiments of that gentleman cannot be considered as evidence that the fifth nerve is either directly or indirectly the nerve which presides over dilatation of the pupil in dogs and cats; nor over contraction of the pupil in rabbits and guinea-pigs.

" 4. In animals in which division of the fifth nerve causes contraction of the pupil it does so by excito-motory action through the sixth nerve, which in these animals supplies the iris in conjunction with the third. The sixth nerve, however, not entering into the formation of the ophthalmic ganglion, which in these animals is extremely small.

"5. In these rodentia, pain of any kind, whether produced by irritation of the fifth, or of any other sensational nerve, will cause more or less contraction of the pupil at the instant, attributable probably to excito-motory action of the iridal fibres of the sixth nerve.

"6. Irritation of the fifth nerve, or of any other sensational nerve, in the cat, and dog, and pigeon, so long as it does not affect the brain to the extent of producing vertigo, *nor the visual sense in any other way*, has no immediate influence over the size of the pupil. Hence, although the fifth is an excitor nerve to the *sphincter iridis* in the rabbit, it is neither directly an excitor nor a motor nerve to it in the dog and cat.

"7. As the third nerve can always, under favourable circumstances, be made to influence the movement of the iris, *immediately* on the application of irritation, and as the iridal portion of the third nerve, in all animals in which the iris is active, passes through the ophthalmic ganglion, it follows that *the ophthalmic ganglion offers no check to the transmission of motor influence along the motor nerve fibres which pass through its substance.*

"8. As irritation of the fifth nerve does not in any animal affect the action of the iris *after* division of the cerebral connections of all the other ocular nerves, and as some portion of the fifth nerve always enters into the ophthalmic ganglion, it follows, *that the filaments of the fifth nerve have not the power of affecting those of the third nerve during their course together through the substance of the ophthalmic ganglion;* or, in other words, *that the lenticular ganglion is not a centre of excito-motory action to the iris.*" P. 25.

Some interesting cases are related with the object of showing that, although the fifth nerve, as we have seen in the above extract, is neither a motor, nor, with some exceptions, an excitor nerve to the iris, yet that it does in some way so affect the internal structures of the eye, as to influence the state of the pupil, inducing under some circumstances contraction, and under others dilatation. The author offers, as a mere hypothesis however, the following as the explanation. "It is allowable to conjecture that the small supply of fifth nerve which enters into the lenticular ganglion, passes through to the retina exclusively; that when in the ganglion, it can affect, during its own conduction of nervous force, the action of the ganglionic vesicles, and consequently call forth the influence, be that what it may, of the ganglionic nerves. Begging the question for the moment, that one effect of the ganglionic influence is the afflux of blood to the part, we can understand how the stimulation of the retina by intense light may cause not only instantaneous contraction of the pupil through the third nerve, but also an altered state of circulation through the retina, through the medium of the fifth nerve, lenticular ganglion and ganglionic nerves, producing painful confusion of sight, or in other words, dazzling and aching in the eye. The unusual supply of blood furnishing at the same time the means of recruiting the over-excited retina. We can understand how one amount of stimulation of the fifth nerve may have such a slight effect on the vascular coat as may merely stimulate the retina, and thus cause contracted pupil (*e. g.* ulcer of cornea, strumous ophthalmia): how a greater amount of stimulation suddenly applied, or the same amount greatly prolonged, may cause so much disorder of the retinal capillaries, as to produce temporary blindness and dilated pupil (*e. g.* severe *tic*). The fifth nerve being in the one case indirectly an excitor of contraction; in the other, of dilatation of the pupil." P. 36.

Proceeding from the consideration of the ganglion of the orbit, the author enters upon a much more extended question—"the real functions of the ganglia." Although it is certain that nutrition, whether normal or abnormal, may be effected independently of a nervous system, as we see in vegetables, yet it is conceived that nervous force is required in animals to regulate and modify the process, by determining the amount of organizable material to be acted on; or, in other words, to determine the quantity of blood that is sent to the organ implicated.

"Modern researches have proved that every proximate constituent of a living body contains, complete within itself, the means of performing its specific action, so long as the requisite conditions are observed. The most important of these conditions is the due supply of organizable material. To regulate the supply according to varying demands, and to ensure order in the discharge of complicated functions which are at once mutually related and yet entirely different, there must be some means of harmonizing various organic actions, and regulating the supply of blood according to the requirements of the different organs. No other system except the nervous presents the characters fitted for effecting this superintendence." P. 47.

It is subsequently proved, according to the author's belief, that the ganglia are the centres which effect this regulation; or that they furnish the true organic nerves. As, however, there is no novelty in this opinion it is proper to state in what the peculiarity of Dr. C. R. Hall's theory consists. He does not believe that the sympathetic concurs in the functions of the vegetative life by exciting the heart's action, or by inducing any contraction of the blood-vessels, or by being necessary to the peristaltic motion of the intestine, for all these phenomena, he contends, are manifested independently of the ganglionic nerves: the influence of the ganglia is, according to the author, exerted on the ultimate molecules of the various organic tissues, and, by modifying the natural affinities possessed by these particles, the act of nutrition may be powerfully affected, and, as a consequence, the action of a muscle, of a nerve, or of a gland. The following passage is the best we have been able to select as illustrative of these views, after taking some trouble to find something more precise and clear.

"As an hypothesis, therefore, capable of explaining all phenomena referrible to the nervous system, so far as physical action is concerned, and apparently open to fewer objections than any other, we infer that the nervous force can stimulate every living molecule endowed with a certain function, to the more active discharge of that function; that it can consequently excite, variously modify, or greatly derange, all vital actions; that muscular contraction, cell-metamorphosis, the evolution of animal heat, are influenced by the nerves in precisely the same manner, the difference in result being due to inherent difference of vital endowment in the molecule subjected to the nervous influence; that the varied fulness and therefore the calibre of the capillary blood-vessels is secondary to, and dependent upon, the activity of the molecular changes going on in their contents, and under the control of the nerves only in so far as these affect the vital actions of the blood." P. 53.

In considering this hypothesis, it is necessary to recollect that the author is here referring to the proper elements of the sympathetic, the cells and vesicles, namely, of its ganglia and the peculiar organic nerves proceeding from them, altogether excluding the white tubular nerves derived from the cerebro-spinal system, and which, it is now well known, although passing through the ganglia and along with the gray filaments originating from those nodules, are in essence foreign to the sympathetic.

We must leave our readers to form their own estimate of the value of Dr. C. R. Hall's opinions, observing, however, that this idea of the nervous force playing in organic matter a part, somewhat similar to that of electricity in inorganic bodies, is not novel; for theories of this kind have, under various names and with different modifications, been often advanced, but never satisfactorily established.

In conclusion, we would, in no unfriendly spirit, advise the author, in any future communication, to be more cautious in advancing mere hypotheses, which, having no legitimate ground for their support, are detrimental rather than serviceable to the real progress of science; and we should further suggest the advantage of greater attention being paid to clearness of language and expression. It would not have been a matter of difficulty, were we so inclined, to point out instances in the present paper in which both these desiderata have been neglected.

